# A dual watermarking scheme for identity protection

**DOI:** 10.1007/s11042-022-13207-1

**Published:** 2022-06-21

**Authors:** Sunpreet Sharma, Ju Jia Zou, Gu Fang

**Affiliations:** School of Engineering, Design and Built Environment, Western Sydney University, Locked Bag 1797, Penrith, 2751 NSW Australia

**Keywords:** Image authentication, Copyright protection, Cybersecurity, DWT-DCT, Halftone, Identity protection, Watermark

## Abstract

A novel dual watermarking scheme with potential applications in identity protection, media integrity maintenance and copyright protection in both electronic and printed media is presented. The proposed watermarking scheme uses the owner’s signature and fingerprint as watermarks through which the ownership and validity of the media can be proven and kept intact. To begin with, the proposed watermarking scheme is implemented on continuous-tone/greyscale images, and later extended to images achieved via multitoning, an advanced version of halftoning-based printing. The proposed watermark embedding is robust and imperceptible. Experimental simulations and evaluations of the proposed method show excellent results from both objective and subjective view-points.

## Introduction

The internet has revolutionised almost every sector of human life. Our dependence on the internet is evident in schools and universities, private organisations and government departments, homes and workplaces. However, the internet has achieved a new prominence in 2020. COVID-19 has severely curtailed the use of offices worldwide forcing many people to work from home, lifting internet usage to new peaks. The internet is invaluable because it is keeping organisations, businesses afloat and people connected to each other during this pandemic, but it also has disadvantages. In this time the hackers are busy too and result of their actions is evident. For instance, in May 2020, a series of identity-related cybersecurity attacks robbed many Australians’ of their pension funds. Thus, in June, the cybersecurity sector was given 1.35 billion Australian dollars to create hundreds of new jobs and ensure Australians’ internet safety [38]. Furthermore, in July, many American celebrities’ and politicians’ Twitter™ accounts were compromised, causing a wave of turmoil amongst social network (SN) platforms [44]. These identity stealing threats are not limited to the electronic media (e-media) but also occur in printed media such as passports, driving licences, identity (ID) cards and postal stamps [45]. These infiltrations can cause a serious damage at personal, societal and national levels, so thwarting them is vital. To this end, watermarking is a tool currently being used to protect various forms of media. This paper aims to present a dual watermarking scheme for images, with potential applications in identity protection, maintaining media integrity and copyright protection in both electronic and printed media.

The watermarking process involves an addition of specific information known as watermark to a medium (host signal) that can later be extracted in order to verify its authenticity [49]. Successful extraction proves the medium’s integrity. The information required at the time of extraction determines whether the watermarking technique is either blind or non-blind. For instance, if the host signal (the original image in this paper) is required at the time of extraction, the process is considered non-blind; otherwise, it is blind. Nevertheless, identity documents such as passports and airport security mechanisms, known as ePassport/smart gates, are non-blind because the embedded watermark generally consists of the owner’s information, in the form of a signature, fingerprint or iris information [8]. These predefined features are matched against those embedded within the document in order to justify its ownership. A positive correlation between the former and latter confirms ownership, whereas a mismatch alarms the border control [17].

At present, both physical/printed and digital identity documents are in use, although the latter are increasingly replacing the former [51, 52]. Nonetheless, manual or physical means of identity verification remain important. For instance, in April 2019, Sydney Airport faced a major IT outage that disabled automated processing mechanisms such as arrival and departure smartgates [2], leaving many passengers stuck in the airport for multiple hours. One way of resolving such issues is to embed identity documents with watermarks of both electronic and physical significance, so that one can be used for verification if other fails. This paper presents a dual watermarking technique that uses both the owner’s bio-metrics (fingerprints) and signatures as watermarks.

In addition to its above-mentioned usage in safeguarding identity documents, the proposed dual watermarking strategy can also be used in several other application scenarios, two of which are presented in this discussion. Firstly, it can be employed by the vaccination certificates. Vaccinating people against COVID-19 is perceived to be an essential part of the pandemic recovery process. Governments worldwide are issuing vaccination passports/certificates that permit fully vaccinated people to enjoy a degree of freedom. Unfortunately, hackers are benefiting by selling fake vaccination passports to the unvaccinated [26, 37]. The proposed dual watermarking scheme can be used to watermark these certificates. Moreover, if there is ever a suspicion and verification is required, the certificate bearer’s signature and fingerprint can then be matched against those embedded within the document. A vaccine certificate is ultimately deemed valid if both embedded watermarks are successfully matched; otherwise, it is invalid. Secondly, many artists nowadays are using social network (SNs) platforms to showcase their art. Unfortunately, these platforms are also the primary source of information leaks and according to Bertini et al. in [6], only one out of thirteen main SNs uses the watermarking technology. Artists can employ the proposed dual watermarking method before uploading the electronic version of their art on these SNs. Thereafter, suppose an artist comes across a stolen version of their work, in which case, the artist can approach the relevant SN. Subsequently, prove the ownership of their work via displaying a successful match of their signature and fingerprint to the ones embedded within the artwork in doubt and ultimately, have the stolen version of their work removed from the internet.

A watermarking scheme is successful when it is able to satisfy three main correlated watermarking requirements: imperceptibility, robustness/security and capacity [49]. A high capacity, that is, space for a large amount of watermark information to be embedded, is desirable. It increases its security/robustness attribute(s), defined by the ability to resist any unauthorised attacks/changes (see [49] for an insight on various attacks). In contrast, growth in capacity adversely affects imperceptibility-the watermark’s ability to stay hidden in the host signal. Attaining an efficient equilibrium between these trade-offs is a well-known challenge in the field of watermarking [49]. The proposed watermarking scheme successfully addresses the aforementioned challenge and the main contributions of the proposed work are described below

### Our contributions

- The proposed watermarking scheme is robust because it uses two distinct watermarks. If one of the watermarks is compromised, there is another to safeguard the medium. This also makes the proposed watermarking scheme high in capacity.
- The proposed method uses a novel median-based coefficient selection procedure. These carefully selected coefficients are modified in equal proportions for the watermark embedding purposes. Such modifications not only conceal the watermark imperceptibly but also uplift the security features of the proposed scheme. The proposed embedding strategy is discussed in detail within the upcoming section on the watermark embedding.
- The proposed watermark embedding is both imperceptible and secure. Consequently, the produced watermarked image gives excellent results in terms of Peak Signal to Noise Ratio (PSNR) and Normalized Cross-Correlation (NCC) values. These matrices are discussed in detail in the experimental results section of this paper.
- The application versatility of the proposed method is tested on both continuous tone/greyscale and multi-tone images. The former is primarily used in electronic media because they can employ the majority shades of the grey palette, whereas the latter is for print media in which the ink/colour pallet is limited.

The rest of this paper is organised in the following manner. Section 2 covers related work in the field, Section 3 discusses the proposed methodology, Section 4 contains experimental results and Section 5 is the conclusion.

## Related work

The term “Digital watermarking” was first used by Andrew Tirkel and Charles Osborne in the early 1990s [59], and since then the process of digital watermarking is proven to be a cornerstone in achieving goals such as media authentication, copyright protection and cybersecurity in general [29]. This section covers the key works on which the proposed watermarking scheme is based.

Firstly, Lin et al. developed a DWT coefficient difference-based watermarking technique [32]. This approach was adopted and widely influenced later works such as [23, 50, 60]. Despite the method’s success in achieving very high imperceptibility with moderate capacity, it struggled with respect to security [39]. Multiple watermarks began to be embedded to address this shortcoming, as described in the most recent literature on watermarking for ID protection or similar applications [4, 8, 17]. Furthermore, the disadvantages associated with methods that are solely based on DWT are restricted if not nullified by pairing them with other techniques such as DCT, singular value decomposition (SVD) and back propagation neural network (BPNN) [20, 25, 35, 53]. However, these hybrid methods have their own flaws. For instance, BPNN or any other machine/deep learning-based technique initially requires an intense amount of computation power and training, making such methods expensive in terms of both resource and time consumption. Hence, integrating multiple techniques into one is a cumbersome task. When compared to other transformations, such as DFT (or its fast version FFT) and DWT, the main merit of DCT-based watermarking methods is their resilience to image compression attacks [12, 41]. Compression is one of the most widely used modifications within the whole image processing space; thus, withstanding it is a must for a robust watermarking strategy. The DCT outperforms the DFT concerning the coefficient energy compaction attribute, making it a better candidate than the latter [24]. A recent review conducted by Begum et al. in [5] has sorted the robustness of the existing transformation techniques as *DCT* > *SV* *D* > *DWT* > *DFT*, justifying the selection of DCT in the proposed method. Moreover, the proposed method is positively influenced by various state-of-the-art methods in [25, 35, 53], which tend to use a combination of DCT and DWT; therefore, the proposed method follows the same trajectory of using both DCT and DWT along with a novel embedding-coefficient selection process.

Secondly, traditional printed documents or printing generally follow the principle of Error diffusion (ED) based halftoning to attain the binary (black and white) equivalent of a greyscale image. In ED, a fixed ratio of quantization error is dispensed amongst the unrefined neighbouring pixels [9]. ED gives out a halftoned image that appears to be homogeneous in tone when viewed from a distance, while maintaining the intensity levels of the input continuous-tone image in its produced binary equivalent. Ordered dithering (OD), on the other hand, is another readily accepted halftoning strategy that compares pixel values of an input greyscale image against a thresholding array known as the “screen”, in order to achieve the output binary image [15]. Traditionally, these two halftoning techniques were widely used in watermarking of binary images/documents because printers were bound by a restricted colour palette. However, modern printers are capable of employing multiple grey tones or colours [34, 46], thus, the scope of traditional printing techniques is also being extended to achieve watermarking in multitoned images for print media.

Recent developments in multi-tone watermarking are discussed in [14, 15, 34, 61] and [63]. These methods include noise-balanced error diffusion (NBEDF), quantization, multiple look-up table (MLUT), direct binary search (DBS) and toggling between two dither array pair, respectively. Although these methods are state-of-the-art and acknowledged within the field, they have several shortcomings. For instance, ED-based methods are known to produce worm-like imprints that ultimately lead to the softening of an image [47], whereas, the prominent visual patterns are present within an image acquired through OD [58]. Such issues adversely affect the fidelity of the watermarked image and reduce its visual quality, thus adding to the image watermarking challenges of both imperceptibility and robustness/security [49]. Some of these issues are addressed in our most recent work that investigates the scope of watermarking in multitoned images [46]. The method is fast and able to achieve high imperceptibility at a decent capacity.

In addition to the aforementioned contributions, the proposed multi-tone watermarking strategy has the following novel aspects which set it apart from its predecessor. First and foremost, the proposed method is a dual watermarking strategy, whereas, our technique in [46] uses a single watermark. Secondly, the experimental results section of this paper covers the analysis of both imperceptibility and robustness, whereas, due to space constraints in [46], it only investigates the imperceptibility aspect. Thirdly, the proposed multi-tone watermarking scheme attains an effective transition amongst the grey levels of a multitoned image while keeping the image information intact, making it effective in the imperceptibility context. Finally, the insertion of an adaptive threshold quantization block in the proposed scheme, boosts its ability to perform over a wide range of grey tones-vital in eradicating any errors that may appear in laborious manual thresholding [32]. The state-of-the-art methods which have positively influenced the proposed scheme are discussed and summarised within Table 1.

**Table 1:** Summary of the related works and comparisons with the proposed method

Methods *↓*	Year	Technique(s)	Imperceptibility	Security	Capacity
Watermarking works for the continuous-tone/greyscale images					
Barr et al. [4]	2019	DWT	Lowest	Lowest	Lowest
Hurrah et al. [20]	2019	DWT+DCT	Medium	High	Medium
Hurrah et al. [21]	2020	DWT	High	High	High
Islam et al. [23]	2020	DWT+SVM	High	Medium	Low
Kamili et al. [24]	2021	DWT+DCT	Medium	Medium	**Highest**
Kang et al. [25]	2018	DWT+DCT+SVD	High	Low	Low
Loan et al. [35]	2018	DWT+DCT	High	Medium	Highest
Sharma et al. [50]	2020	DWT	Hgh	Medium	Low
Singh et al. [53]	2018	DWT+DCT+BPNN	Low	Medium	High
Verma et al. [60]	2015	DWT	Medium	Medium	Low
**Proposed**	2021	DWT+DCT	**Highest**	**Highest**	High
Watermarking works for the Multi-tone images					
Chan et al. [9]	2019	ED+Quantization	High	Lowest	Low
Chen et al. [10]	2018	ED+Toggling	High	Medium	**Highest**
Guo et al. [14]	2010	NBDEF	Medium	Medium	High
Guo et al. [15]	2018	OD+MLUT	Lowest	Medium	Low
Guo et al. [16]	2019	ED+OD	Medium	High	Medium
Lee et al. [31]	2016	OD	High	Low	Low
Sharma et al. [50]	2020	OD+Quantization	High	High	Medium
Xu et al. [61]	2019	DBS	High	Medium	Medium
**Proposed**	2021	OD+Quantization	**Highest**	**Highest**	High

## Methodology

**Fig. 1:**
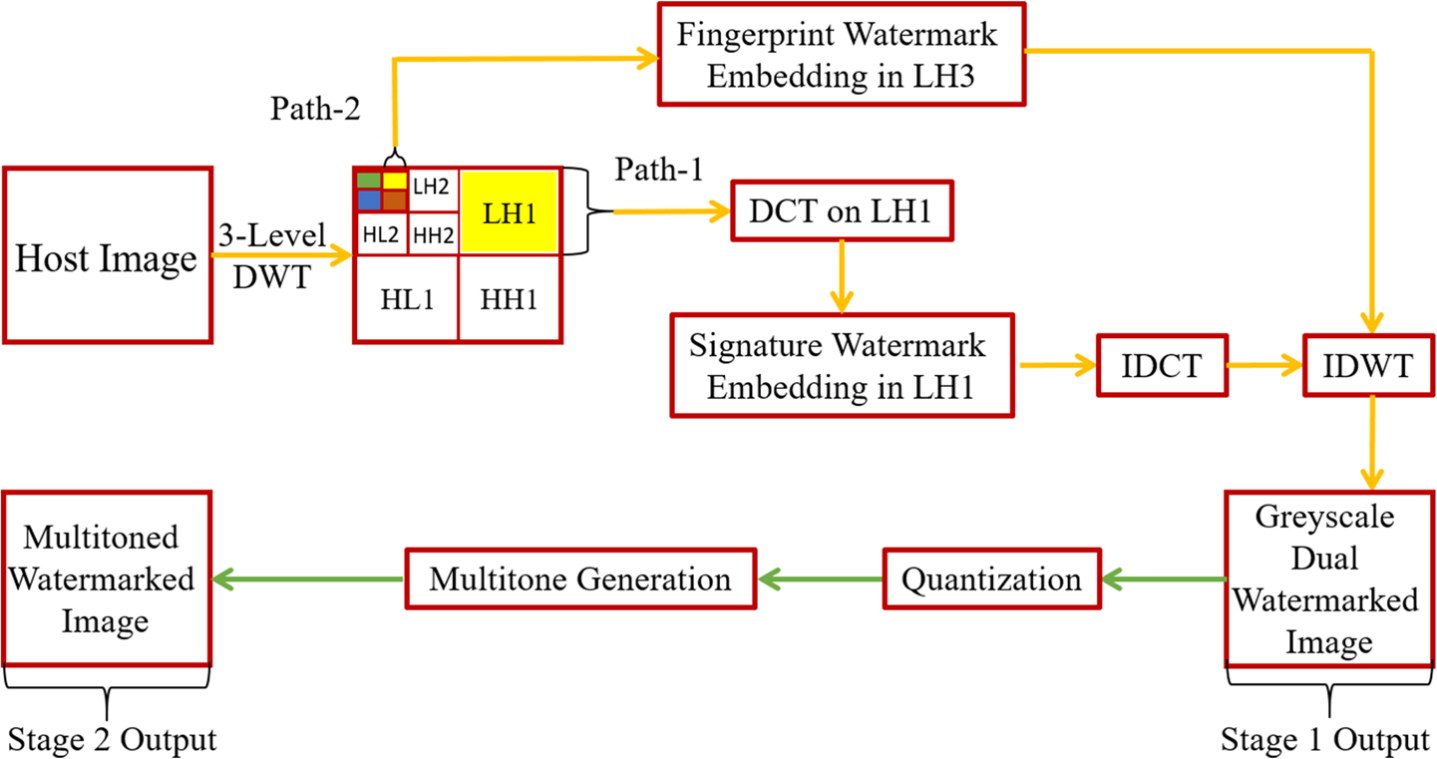
Blueprint of the proposed method. The yellow arrows represent the steps involved within stage-1, whereas, the green arrows are for stage-2 steps

An overview of the proposed watermarking scheme that is divided into two stages is given in Fig. 1. Stage-1 corresponds to the dual watermarking of greyscale image(s), and stage-2 transcends the output of stage-1 to its printable multitoned representation. Stage-1 is further divided in two paths: path-1 consists of the steps involved in the signature watermark embedding, and is inspired by the literature in [20, 25, 35, 53]; path-2 focuses on fingerprint watermark embedding, which is influenced by techniques used in [23, 32, 60]. In path-1, the host image/original signal of size *m* x*n* (512 × 512 in this paper) is decomposed into wavelet coefficients by using DWT [32]. Note, as in [23, 32, 60], a three-level decomposition using DWT is selected in the proposed method, so that a fair comparison between the two can be established. Another crucial notion to be acknowledged is that the proposed method serves the host image with dimensions in the power of two, otherwise the host image is resized in order to adjust its dimensions to the closest power of two. Such adjustments are required to make sure that the wavelet subbands extracted as a result of DWT are of similar dimensions, an important requirement to achieve multi-resolution analysis (MRA). A detailed discussion on MRA and resizing is presented in [13].

**Fig. 2:**
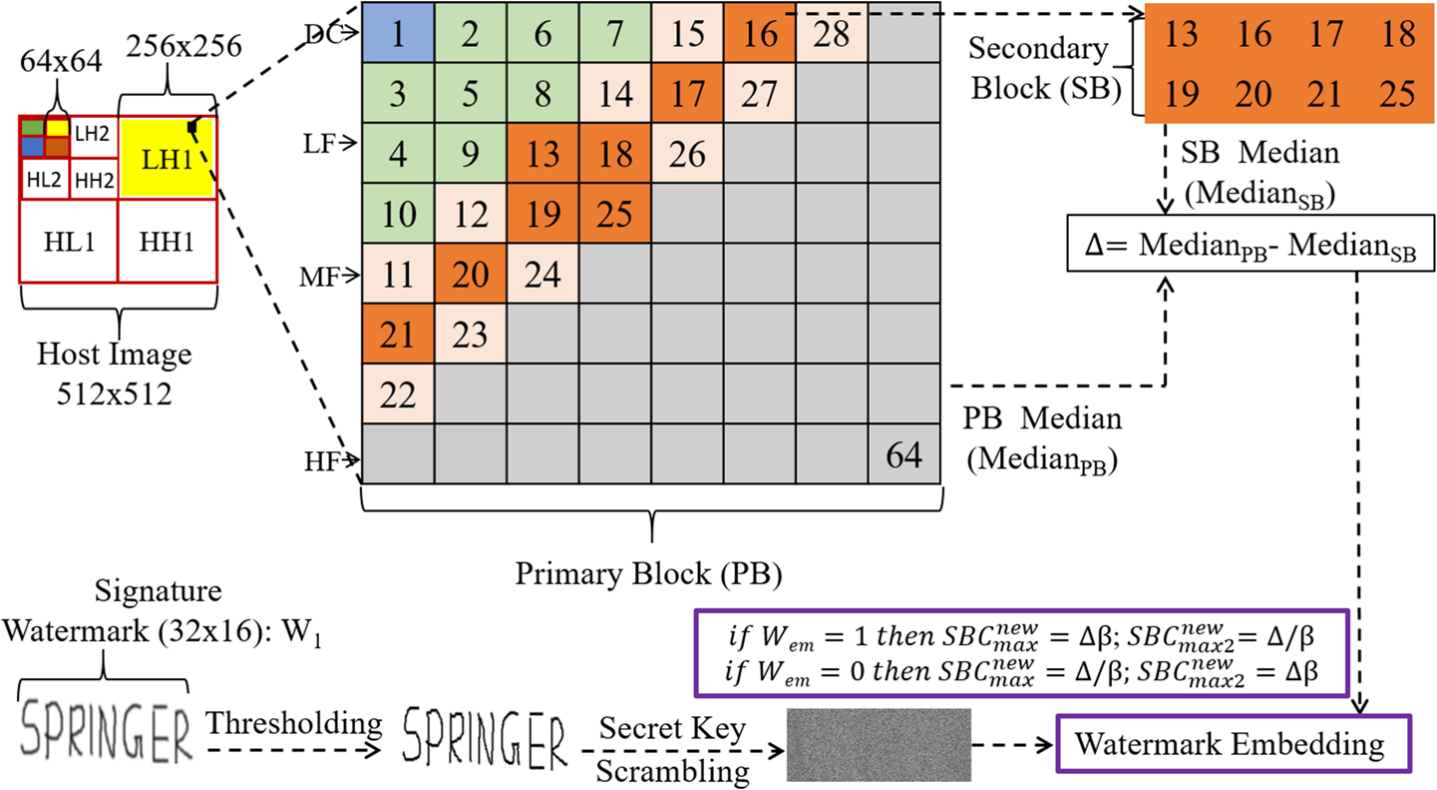
Path-1: Signature watermarking process. Digits within the Primary Block (PB) are the numbers allocated to DCT coefficients, where DC being the lowest frequency component, is labelled as 1 and 64 is dedicated to the highest frequency component

**Fig. 3:**
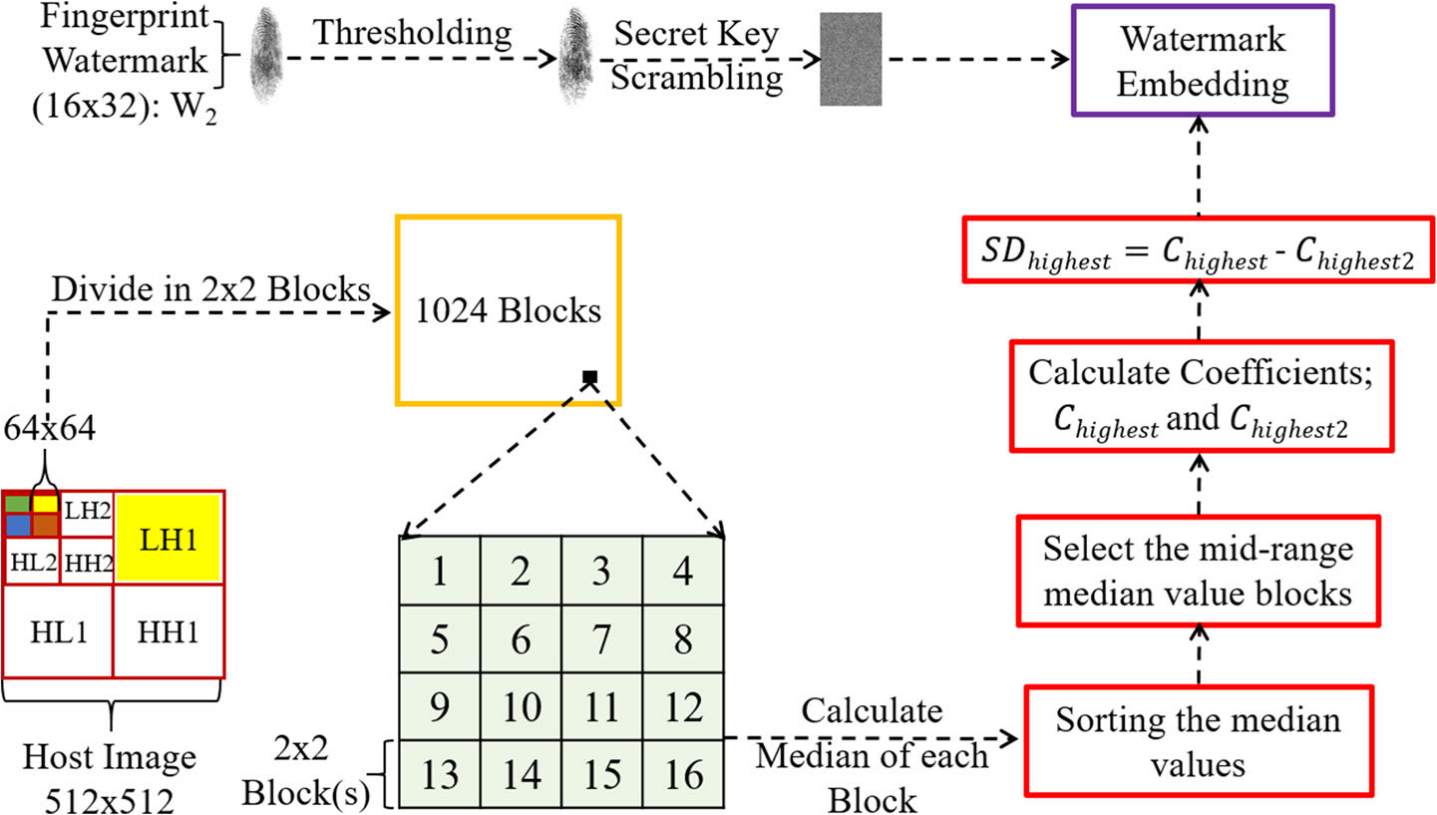
Path-2: Fingerprint watermark embedding process. Digits within the light green coloured block(s) correspond to a 2x2 block

A single-level DWT decomposition of an image produces four wavelet subbands, one of which is called the approximate subband and the remaining three as detail subbands. The approximate subband *LL* is composed entirely of low-frequency coefficients, whereas one of the detail subbands (*HH*) is solely made up of high-frequency components, and others (*LH* and *HL*) contain both high and low-frequency wavelet coefficients, respectively. Similarly, a three-level DWT generates one approximate subband *LL3* (represented by the solid green colour in Fig. 1) and nine detail subbands (*LH1-LH3, HH1-HH3, HL1-HL3*). As *LL* is comprised of low-frequency components, it represents the majority of the image information and can easily induce deformities on alterations, therefore is not recommended for watermark embedding. Subsequently, adding watermark bits to *HL*3 and *HH*3 (represented as the solid orange and blue colour blocks in Fig. 1) is not ideal because the watermark can easily be breached through attacks such as rotation or lossy compression [49]. Thus, *LH1* and *LH3* subbands (both depicted by the solid yellow coloured blocks in Figs. 2 and 3) are selected for signature and fingerprint watermark embedding, respectively. An in-depth study of the behaviour of embedding watermark bits in each of these subbands is documented in [23]. The upcoming subsections on watermark embedding in this paper cover the rest of the proposed watermarking strategy.

### Stage-1: Signature watermark embedding (Path-1)

Firstly, the *LH*1 subband of size 256x256 is divided into 8x8 blocks, a total of 1024 non-overlapping blocks. A magnified illustration of such a block is highlighted in Fig. 2. Subsequently, DCT is carried out on an 8x8 block, made up of *LH*1-DWT coefficients, in order to yield their respective DCT coefficients, and collectively they form a primary block. The block itself is termed as the primary-block (PB) in Fig. 2. Based on frequencies, DCT coefficients are categorised as low-frequency (LF), mid-frequency (MF) and high-frequency (HF) and the very first low-frequency coefficient is known as the direct-current (DC) coefficient. MF coefficients are chosen for signature watermark embedding because these coefficients, unlike their counterparts (LF and HF coefficients), allow alterations while maintaining a suitable balance between imperceptibility and robustness. A full account of the behaviour of DCT coefficients can be found in [41]. Similar to [25], a secondary-block (SB) is constructed by eight of the total MF coefficients in a PB and their allocated position numbers in Fig. 2 are 13,16 − 21,25. Subsequently, the median value of the PB is calculated and labeled as *Median*_*PB*_ (primary-block median) and the secondary block with *Median*_*SB*_ (secondary-block median). Thereafter, the difference between the former and the latter is calculated and depicted by Δ (see [35] for median calculations).

Secondly, the signature watermark (*W*_1_) of size 32x16 (for illustration purposes in this paper) is prepared by a series of steps as shown in Fig. 2. The very first step involves the binarization of the selected watermark by thresholding it to a value of 128. This limits the watermark to only two pixel values of 0 (Black) and 255 (White), corresponding to 0 and 1 in binary, respectively. Subsequently, the binarized watermark is scrambled using a secret key. The purpose of using such a key is to maintain the integrity of the medium during transmission, because the very same key is required at the time of validation through the watermark extraction process (discussed later in this paper). The Fisher–Yates shuffle algorithm is used throughout the proposed watermarking method, due to its robust performance and state-of-the-art usage (see [60] for more details of this shuffling concept). Note, the aforementioned steps in the signature watermark preparation are identical for the fingerprint watermark preparation, which is discussed in detail in the upcoming subsection. Once the signature watermark is prepared and the value of Δ is calculated, the maximum (*SBC*_*max*_) and the second-maximum (*SBC*_*max*2_) valued coefficients within the SB are modified in order to meet the following criteria. If the watermark bit to be embedded (*W*_*em*_) in the SB is 1, then,

1 $$ \begin{array}{@{}rcl@{}} {SBC_{max}}={\Delta}\beta; {SBC_{max2}}={\Delta}/\beta \end{array} $$

and if it is 0, then,

2 $$ \begin{array}{@{}rcl@{}} {SBC_{max}}= {\Delta}/\beta; {SBC_{max2}}={\Delta}\beta \end{array} $$

where *β* stands for the modification factor. Similar to [35, 41], *β* is selected to be 0.5 in this paper, which is also in line with [25]. The main advantage of the proposed embedding strategy (represented by the purple boundary in Fig. 2) is that the coefficient modifications are carried out in pairs in equal proportions, thus, safeguarding the medium against non-geometrical attacks, such as unwanted compression. The adopted coefficient modification in reality is a coefficient scaling procedure, therefore, if one of the coefficients is scaled up by a factor of *β* the other coefficient must be scaled down by the same factor. Consequently, the median value of the PB is kept intact and so is the overall imperceptibility. Furthermore, any unauthorised change would cause a shift in the median values, degrading the appearance of the final watermarked image, thus confirming a security breach. Finally, the aforementioned steps are carried out on the rest of the predefined 8 × 8 blocks within the *LH*1 subband. This represents the culmination of path-1 of Stage-1 and thus the signature watermark embedding.

### Fingerprint watermark embedding (Path-2)

The decomposition level(s) of DWT is chosen with respect to the sizes of the watermark(s) and the host image. The following scenarios are used to further expand on this discussion. Firstly, when the dimensions of the host image are 512 × 512 and that of the fingerprint watermark are 32 × 16 or 32 × 32, the *LH3* subband is selected over the *LH2* subband. This is because the latter is outperformed by the former with respect to optimising the correlation between imperceptibility and robustness. Notwithstanding, the literature in [62, 65], which highlights the higher the decomposition level, the higher the robustness. The *LH4* subband, produced due to the four-level DWT decomposition, can still not be employed in this scenario because the *LH4* subband would be too small to accommodate the fingerprint watermark, equal to or larger than 32 × 16 or 32 × 32 in size. Similarly, the decomposition levels higher than three are unsuitable for embedding the fingerprint watermark of such dimensions. Note, the capacity calculations of the proposed watermarking scheme are presented below within Section 4.2. It is also recommended not to reduce the watermark size because the smaller the watermark, the harder it is to verify. Therefore, the *LH3* is preferred over the *LH2* in this scenario. Secondly, when the dimensions of the host image are 256 × 256 and that of the fingerprint watermark are 32 × 16 or 32 × 32, the *LH2* subband is selected. This is because the subbands produced at three or higher levels of the DWT decomposition of the host image: 256 × 256 in size are not big enough to accommodate a fingerprint watermark of dimensions 32 × 16 or 32 × 32. Although images that are 256 × 256 in size are hardly used nowadays, such embodiments can still employ the proposed method, and the *LH2* subband can be used in such a scenario. Third, when the dimensions of the host image are 1024 × 1024 and that of the fingerprint watermark are 32 × 16 or 32 × 32, either *LH3* or *LH4* subband can be selected for the watermark embedding. However, the literature in [62, 65] also highlights that the higher the decomposition level, the higher the processing time; therefore, the *LH4* is not preferred for real-time applications. Henceforth, the proposed method’s selection of the DWT decomposition levels is guided by the discussions above. Moreover, for illustration purposes, the first scenario out of all the scenarios mentioned above justifies the choice of the *LH3* subband for the fingerprint watermark embedding. Finally, the selection of the *LH3* subband in path-2 of the proposed method is also inspired by techniques in [23, 32, 60]. Each of these methods has employed the *LH*3 subband for the watermark embedding; however, the proposed method is different in the embedding block selection procedure(s) to the ones adopted by the authors in [23, 32, 60] in the following ways.

Methods in [23, 32, 60] rely on three or more secret keys. Notwithstanding the success of utilising multiple keys in achieving robustness, they tend to require high computational power, whereas the proposed method uses only one secret key (as discussed above) throughout. Additionally, the security/robustness aspect can be bridged by inserting multiple watermarks; their use in the proposed method also leads to high capacity. Finally, the block selection procedure is performed through shuffling on multiple occasions using multiple keys in [23, 32, 60]. It is not optimal for transmission because all of these keys are required at the time of watermark extraction and this can cause problems if transmission bandwidth is limited. This limitation associated with block selection is addressed below.

In Fig. 3, *LH*3 coefficients are divided into a total of 1024 non-overlapping blocks, each 2x2 in size. The median value of each of these blocks is calculated and the individual values are collectively sorted either in an ascending or descending order. Then, 512 of these median values falling within the mid-range of the total 1024 values are selected and so are their 512 corresponding 2x2 blocks. Each of these selected 512 blocks are tagged as *M*_*b*_ blocks in the proposed method. Subsequently, the difference amongst the highest valued (*C*_*highest*_) and the second-highest valued (*C*_*highest*2_) coefficient is quantified and labelled as the *highest significant difference* (*SD*_*highest*_). This series of steps is iterated for each of the 512 *M*_*b*_ blocks and the fingerprint watermark (*W*_2_) of size 16x32 (512 bits) is prepared by following the same sequence of steps as performed above within path-1. Thereafter, the embedding process is commenced by the *embedding quantizer* (*EQ*). The *EQ* (represented by the purple boundary in Fig. 3) quantizes the highest valued coefficient (*C*_*highest*_) of a corresponding *M*_*b*_ block to $C^{new}_{highest}$ by using (3) and (4).

If the watermark bit is 0, the significant difference is quantized to zero by equalising the highest and the second-highest coefficient.

3 $$ \begin{array}{@{}rcl@{}} {C^{new}_{highest}}=C_{highest2} \end{array} $$

If the watermark bit is 1,

4 $$ \begin{array}{@{}rcl@{}} {C^{new}_{highest}}=\left\{\begin{array}{cc} {C_{highest}+T}, &    if (SD_{highest})< max(\lambda,T) \\ {C_{highest}}, &    otherwise \end{array}\right. \end{array} $$

In (4), *T* is the threshold value used for quantization. Indistinguishable from [60], *T* = 11 is adopted to be the threshold value for the proposed method as well. The empirical selection procedure of *T* is detailed in [32]. The *average significant difference* of all *M*_*b*_ blocks is represented by *λ*, which is quantified by using (5).

5 $$ \begin{array}{@{}rcl@{}} \lambda = \left\lfloor{\frac{{\sum}^{M_{b}}_{k=1}SD^{k}_{highest}}{M_{b}}}\right\rfloor \end{array} $$

Subsequent steps in the overall proposed dual watermarking scheme include the merging of modified coefficient blocks with unaltered blocks of both *LH3* and *LH1* subbands and performing the inverse of both DCT and DWT (IDCT-IDWT), respectively. Consequently, a final dual watermarked image comprising of both signature and fingerprint watermarks is achieved using (6).

6 $$ \begin{array}{@{}rcl@{}} WI_{Final}=HI_{Original}(1+\alpha W_{Total}) \end{array} $$

where *WI*_*Final*_, *HI*_*Original*_, *W*_*Total*_ and *α* stand for the final watermarked image, original host image, total watermark embedded and the watermark strength parameter, respectively. The value of *α* ranges between “0” and “1” and it defines the visibility of the inserted watermark, with 1 being fully visible and 0 as invisible [7]. It is established empirically that the proposed scheme yields the best results when *α* is between [0.3-0.5]. Similar to methods in [60] and [23], *α* = 0.4 is chosen for the experimental simulations for the proposed method.

### Stage-2: Multi-tone generation

**Fig. 4:**
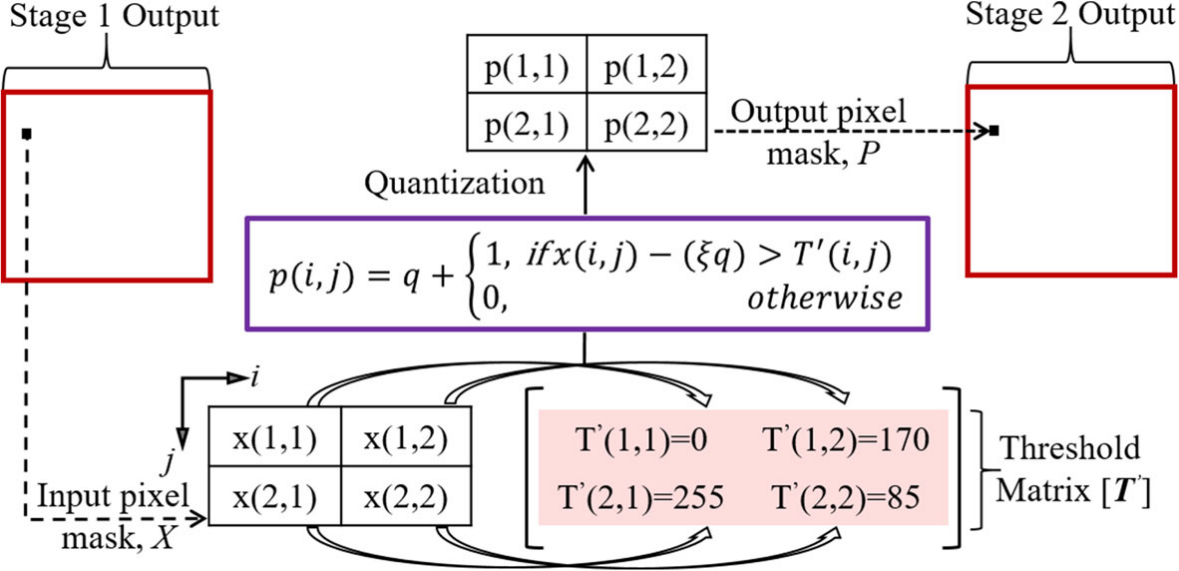
Overview of the proposed multitoning process

Stage-1 of the proposed method deals with a continuous-tone/greyscale image consisting of 256 grey tones, whereas stage-2 deals with an image(s) that is made up of fewer grey tones. When a continuous-tone/greyscale watermarked image is processed by an application that can only handle a limited set of grey tones, such as printing, the embedded watermark gets damaged or deforms. Stage-2 of the proposed method can manage such watermarked images more effectively and provides more immunity to the embedded watermark(s) when compared with state-of-the-art methods. The quantization strategy proposed within stage-2 restricts the embedded binary watermark bits from changing their states; the white embedded bit stays white, whereas the black bit stays black. Consequently, the proposed method performs effortlessly over a broader range of multiple grey tones without damaging the embedded watermarks. Bayer’s OD approach is both robust in execution and cheap in computational complexity [15], thus, it is a component of the stage-2 procedure of the proposed method. Primarily, the implementation of OD requires comparing a 2-D pixel template of an input image against a 2-D thresholding mesh called the threshold matrix or screen (depicted by the pink colour block in Fig. 4.

It is vital to acknowledge that the input image in stage 2 is the *output* of stage 1 in the form of a continuous-tone watermarked image. During this comparison, pixel values of the input image mask are quantized as per their corresponding threshold values within the thresholding grid. In order to differentiate between the thresholding symbol of stage 1, depicted by *T*, the stage 2 thresholding elements are described by $T^{\prime }$. The element values of the thresholding matrix [***T***$^{\prime }$] are both constant and different from each other. As per Bayer, if threshold matrices are in powers of two, an optimal dither pattern can be achieved, thus leading to the pattern noise being as high in frequency as possible [48], which is desirable in the context of imperceptibility because the human visual system (HVS) is less sensitive to high-frequency modulations.

In a greyscale image, effective quantization relies on the total number of grey levels to be quantized, as well as the size of the threshold matrix [58]. Stage-2 of the proposed method is verified over a range of grey levels i.e. 4, 8 and 16, moreover, a 2x2 threshold matrix is adopted, in line with methods in [46] and [48]. Another reason for this choice is that our preceding works on multitoning incorporated the Floyd-Steinberg ED kernel because it scatters the quantization error amongst four neighbouring pixels; therefore, the adopted thresholding matrix [***T***$^{\prime }$] is composed of four pixels [47]. The selected thresholding matrix is based on the dispersed-dot pattern, which is discussed in-depth by [58].

The element values of matrix [***T***$^{\prime }$], as shown in Fig. 4, execute the proposed multitoning process at four grey levels, where or at any other instance in this paper *i* and *j* stand for the row and column locations, respectively. Quantization at four levels ranging from [0 − 3] can be achieved by splitting the total intensity levels (255, starting with zero) into three equal intervals of 85 each. The correlation between quantization levels (*q-levels*) and input pixel intensities is formulated as per (7).

7 $$ \begin{array}{@{}rcl@{}} {p(i,j)}=q+\left\{\begin{array}{cc} {1}, &    if x(i,j)-(\xi q)> T^{\prime}(i,j) \\ {0}, &    otherwise \end{array}\right. \end{array} $$

Figure 4 gives a visual representation of the working of (7), where *x*(*i*,*j*) is the input pixel value belonging to a 2x2 input pixel mask (*X*, see Fig. 4) and it is quantized to obtain the output pixel value, *p*(*i*,*j*) belonging to a 2x2 output pixel mask (*P*).

**Fig. 5:**
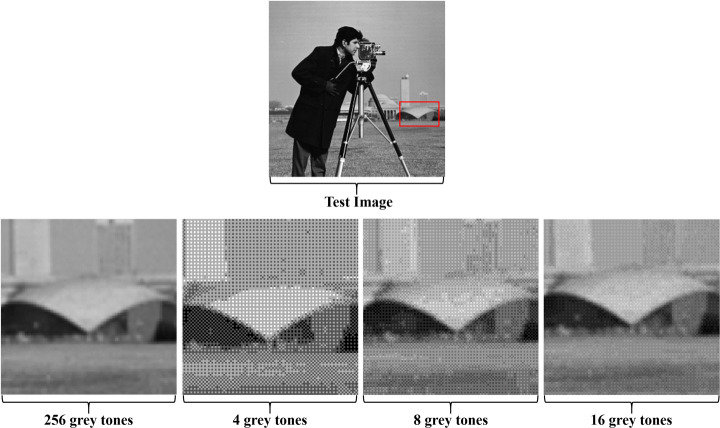
Working illustration of the proposed multitoning process

The value of *ξ* in (7) is the integer that defines the *q-level*, as such, $q=\left \lfloor {x(i,j)/\xi }\right \rfloor $. Consequently, manipulations in the value of *ξ* can achieve the desired quantization amongst the grey levels, thus, producing an imperceptible image even from a limited set of grey tones or colour palette. For instance, 255/37 = 7 and 255/17 = 15 can successively harvest images comprised of 8 (former) and 16 (latter) grey levels. However, *ξ* = 85 is stipulated for further discussion in this paragraph because 255/85 = 3, generates 4 grey levels, [0 − 3]. Moreover, *q* in (7) is defined by a floor function (⌊.⌋), hence, the product, *ξq*, at any given instance is limited to only one of the four possible values of 0, 85, 170 and 255, thus ultimately producing an image with 4 grey tones. Subsequently, such a quantization restricts the embedded binary watermark bits from changing their states; that is, the white embedded bit stays white and the black bit stays black. Consequently, as demonstrated in Figs. 5, 9 and 10, the proposed method performs effortlessly over a wider range of multiple grey tones without damaging the embedded watermark. Lastly, the culminating watermarked image is extracted by processing every pixel of the stage-2 input image by using the quantizer based on (7).

### Watermark extraction

Non-blind watermarking requires the host and the watermarked signals at the time of extraction. Non-blind extraction in the spatial domain can be achieved by using (8). It is essential to realise that (8) only outputs the watermark(s) in a scrambled state. Thus, unscrambling it is the last step of the extraction process, which is achieved by executing an inverse of the aforementioned secret key.

8 $$ \begin{array}{@{}rcl@{}} W_{Total}={\frac{WI_{Final}-HI_{Original}}{\alpha HI_{Original}}} \end{array} $$

When employed by electronic media, watermarks from a grayscale/continuous tone image can be extracted at the end of stage-1, using the proposed watermark extraction process. Similarly, when employed by print media, watermarks from a multi-toned image can be extracted at the end of stage-2. Note, as the proposed embedding strategy is implemented in the frequency domain, the relevant extraction process can therefore only be executed in the frequency domain as well. A step-by-step breakdown of the employed extraction process is provided in Algorithm 1. 
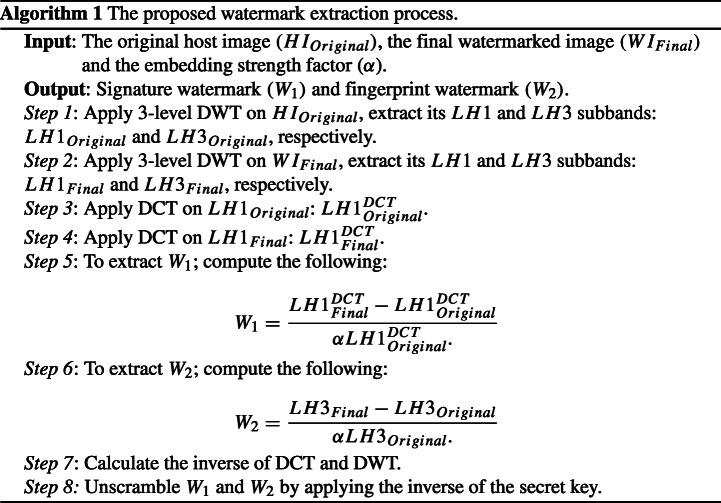


## Experimental results

**Fig. 6:**
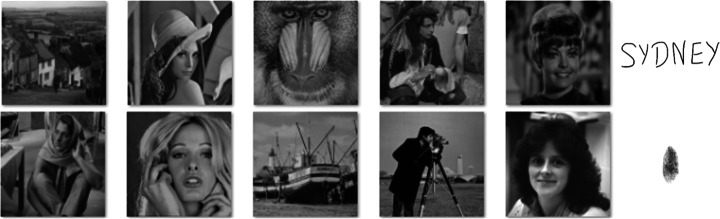
Test images (publicly available at [11] and [57]) and original watermarks used for illustrations in this paper. First row (left to right): Goldhill, Lena, Baboon, Pirate, Zelda and signature watermark. Second row (left to right): Barb, Tiffany, Boat, Cameraman, Lady and fingerprint watermark

The versatility of the proposed watermarking scheme is tested on 96 greyscale images. Image datasets are publicly available at [11] and [57]. Figure 6 shows 10 examples of the total test images.

The experiments are conducted on a machine with i7-8650U CPU running at 1.9 GHz, 16 GB RAM and a 64-bit operating system using MATLAB (R2021a). Note, the experimental analysis presented in this paper is conducted on images as small as 128x128 and as large as 2048x1152 in pixel resolution. Statistically, the experimental simulations were run 25 times using the aforementioned machine. The average results of these simulations are presented in Tables 2, 3, 4 and 5. To this end, the proposed watermarking scheme is stable and achieves confidence of 98% in PSNR, NCC and processing time values related to both the continuous-tone and multi-tone images. In terms of execution, the proposed scheme works 100% in watermarking and multi-tone generation processes, respectively.

**Table 2:** Imperceptibility and capacity analysis. The average PSNR values are in decibels (dB) and the capacity is measured in the total number of bits embedded within the host image. Note, N/A in this table or at any other instance in this paper stands for “Not Available”

Continous-tone/ Greyscale images						
**Methods** →	Method [4]	Method [20]	Method [35]	Method [53]	Method [24]	Proposed
**PSNR**→	37.24	40.45	41.53	37.84	41.0	**46.53**
**Capacity**→	1000 ∣ *Lowest*	4096 ∣ *Medium*	4096 ∣ *Medium*	4096 ∣ *Medium*	**8192** ∣ ***Highest***	5120∣ *High*
Multi-tone images						
**Grey level***↓*	**Methods** →	Method [9]	Method [15]	Method [63]	Method [46]	Proposed
4	**PSNR**→	41.6	40.8	41.2	41.9	**42.9**
8	**PSNR**→	44.9	N/A	44.6	45.2	**46.97**
16	**PSNR**→	48.7	N/A	48.3	48.9	**50.53**
	**Capacity**→	2048 ∣ *Low*	2048 ∣ *Low*	4096∣ *Medium*	2048 ∣ *Low*	**5120**∣ *High*

**Table 3:** Robustness/Security analysis of the extracted watermark(s) from the continuous-tone images and its comparison with state-of-the-art blind and non-blind watermarking methods

Blind watermarking methods					
Attacks *↓* /Methods →	Method [4]	Method [20]	Method [35]	Method [53]	Method [28]
Attack-free/ NCC→	1	1	1	1	1
Rotation 45^∘^	**0.98**	0.94	0.96	0.908	0.841
Median filtering (3 × 3)	N/A	0.966	0.925	0.87	**1**
Gamma correction at (*γ* = 0.50)	N/A	N/A	N/A	N/A	N/A
Salt & Pepper noise (0.02)	N/A	0.960	0.854	0.867	0.991
Gaussian noise (0.001)	N/A	**0.99**	0.9179	0.818	0.951
Histogram equilization	N/A	N/A	0.961	0.902	**1**
Blurring (5%)	N/A	N/A	N/A	N/A	0.937
Sharpening (25%)	N/A	0.975	0.957	N/A	N/A
Scaling (50%)	0.66	0.99	0.987	0.843	**1**
Compression (QF= 40)	N/A	0.931	0.926	0.981	0.669
Compression (QF= 50)	N/A	0.944	0.932	0.983	N/A
Compression (QF= 60)	N/A	0.967	0.954	0.985	0.754
Average NCC →	0.88	0.966	0.943	0.916	0.904
Non-blind watermarking methods					
	Method [33]	Method [3]	Method [1]	Method [50]	**Proposed**
Attack-free/ NCC→	1	1	1	0.98	**1**
Rotation 45^∘^	0.81	0.94	0.96	0.96	**0.98**
Median filtering (3 × 3)	0.87	0.989	0.949	0.91	0.97
Gamma correction at (*γ* = 0.8)	N/A	0.994	0.949	0.952	**0.96**
Salt & Pepper noise (0.02)	0.8	**0.997**	0.924	0.964	0.971
Gaussian noise (0.001)	0.79	0.944	0.858	0.95	**0.99**
Histogram equilization	N/A	0.987	0.97	0.94	0.974
Blurring (5%)	N/A	N/A	N/A	0.92	**0.983**
Sharpening (25%)	N/A	0.948	0.889	0.97	**0.986**
Scaling (50%)	N/A	0.988	0.94	0.93	**0.99**
Compression (QF= 40)	0.87	N/A	N/A	0.95	**0.988**
Compression (QF= 50)	0.9	0.99	0.96	0.967	**0.991**
Compression (QF= 60)	0.92	N/A	N/A	0.986	**0.996**
Average NCC →	0.89	0.975	0.933	0.949	**0.983**

**Table 4:** Robustness/Security analysis of the extracted watermark(s) from the multi-tone images

Methods →	Method [15]			Method [46]			Proposed		
Attacks *↓*/Grey level →	4	8	16	4	8	16	4	8	16
Attack-free/ NCC→	0.904	N/A	N/A	0.912	0.932	0.952	0.936	0.958	0.981
Rotation 45^∘^	0.884	N/A	N/A	0.891	0.915	0.934	0.928	0.948	0.974
Gamma correction (*γ* = 0.75)	0.837	N/A	N/A	0.861	0.885	0.912	0.887	0.932	0.957
Gamma correction at (*γ* = 0.50)	0.852	N/A	N/A	0.876	0.893	0.918	0.894	0.938	0.962
Salt & Pepper noise (0.02)	0.876	N/A	N/A	0.901	0.916	0.928	0.926	0.941	0.968
Gaussian noise (0.001)	0.872	N/A	N/A	0.904	0.924	0.931	0.928	0.945	0.971
Histogram equilization	0.868	N/A	N/A	0.896	0.917	0.926	0.921	0.934	0.958
Blurring (5%)	0.874	N/A	N/A	0.906	0.922	0.934	0.918	0.939	0.962
Sharpening (25%)	0.869	N/A	N/A	0.905	0.916	0.926	0.913	0.940	0.967
Compression (QF= 40)	0.834	N/A	N/A	0.886	0.904	0.918	0.915	0.931	0.938
Compression (QF= 50)	0.851	N/A	N/A	0.902	0.911	0.923	0.922	0.941	0.946
Compression (QF= 60)	0.877	N/A	N/A	0.905	0.923	0.931	0.926	0.944	0.962
Average NCC →	0.867	N/A	N/A	0.895	0.913	0.928	**0.918**	**0.941**	**0.964**

**Table 5:** Processing time evaluations for a 512 × 512 image

Continuous-tone/ Greyscale images						
Time (Sec.)	Method [4]	Method [54]	Method [24]	Method [27]	Method [55]	Proposed
*Time*_*Embedding*_	90	6.8	**3.3**	N/A	6.1	5.9
*Time*_*Extraction*_	55	4.8	**1.5**	N/A	4.2	3.1
*PT*_*Continuous*_	145	11.6	**4.8**	19.2	10.3	9.0
Multi-tone images						
Grey level *↓*∣${Methods}\rightarrow $		Method [9]	Method [15]	Method [63]	Method [46]	Proposed
4$\mid Time_{Multitoning} \rightarrow $		4.632	**4.495**	4.753	4.593	4.6
8$\mid Time_{Multitoning} \rightarrow $		4.962	N/A	5.134	**4.727**	4.8
16∣*Time*_*Multitoning*_→		5.496	N/A	5.663	**5.046**	5.10

### Performances matrices and baseline

A quantitative evaluation of the proposed method in terms of imperceptibility and capacity, using the continuous tone/greyscale and the multi-tone images, is contained within Table 2. The robustness/security analysis presented in Table 3 compares the effect of various attacks on the continuous-tone watermarked image achieved by the proposed method to other state-of-the-art methods such as [4, 20, 24, 35, 53]. Similarly, Table 4 compares the same effect on the watermarked multi-tone image produced by the proposed method, operating at different grey tones, against the ones delivered by the OD-based methods in [15, 46] and the ED-based methods in [9, 63], respectively. The imperceptibility is measured in decibels through PSNR, given by (9). A high PSNR value indicates high imperceptibility. The average of PSNR results are summarised in Table 3.

9 $$ \begin{array}{@{}rcl@{}} {PSNR}=10\log_{10} \frac{(2^{d}-1)^{2}wh}{{\sum}_{i=1}^{w} {\sum}_{j=1}^{h}(z[i,j]-p[i,j])^{2}} \end{array} $$

where *d* is the bit depth of a pixel, *w* and *h* are the image width and height, respectively. Furthermore, *z*(*i*,*j*) and *p*(*i*,*j*) indicate pixel values of the host/original continuous-tone image and the watermarked image produced as a result of the dual watermarking operation, respectively. Similarly, in the case of a multi-tone image, *z*(*i*,*j*) belongs to the multitoned image *without* the watermark, whereas, *p*(*i*,*j*) corresponds to a pixel local within the watermarked multitoned image. Subsequently, the robustness of the proposed method is tested through NCC, given by (10), where *W* and $W^{\prime }$ stand for the original and extracted watermarks of dimensions *P*_*w*_ and *Q*_*w*_, respectively.

10 $$ \begin{array}{@{}rcl@{}} {NCC}=\frac{{\sum}_{i=1}^{P_{w}} {\sum}_{j=1}^{Q_{w}}(W[i,j]\times W^{\prime}[i,j])}{\sqrt{{\sum}_{i=1}^{P_{w}} {\sum}_{j=1}^{Q_{w}}(W^{2}[i,j])}\times {\sqrt{{\sum}_{i=1}^{P_{w}} {\sum}_{j=1}^{Q_{w}}(W^{'2}[i,j])}}} \end{array} $$

The NCC values ought to have a range between [0 1], with 0 being the least in similarity and 1 being the highest. A further insight on NCC and its theoretical basis can be gained from [40, 64]. Moreover, NCC’s usage in state-of-the-art watermarking works is outlined within the recent surveys in [36] and [56].

**Fig. 7:**
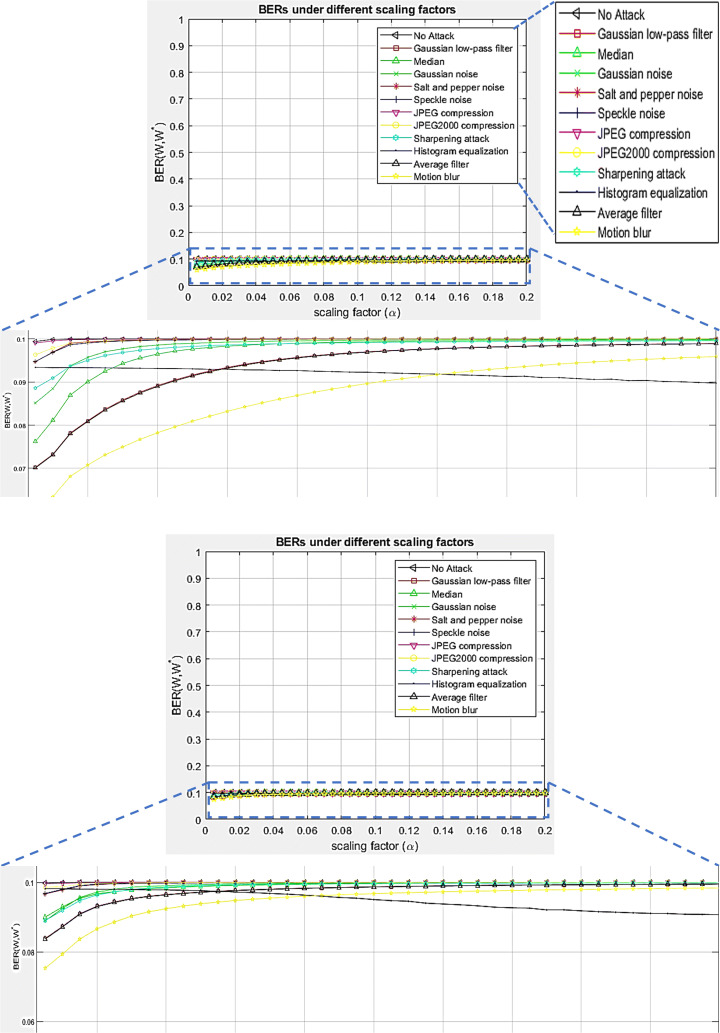
BER vs. scaling factor (*α*) plots illustrating the watermark extraction error rate under the influence of different attacks. *Top*: BER values of the extracted fingerprint watermark. *Bottom*: BER values of the extracted signature watermark. Best viewed when zoomed in

The watermark extraction error is a factor of the watermark embedding strength factor (*α*), also known as the scaling factor and the type of watermarking attack. Overall, the extraction process is stable with a maximum error rate of 3%, illustrated by Fig. 7. The error rate is calculated as the bit-error-rate (BER), by using (11);

11 $$ \begin{array}{@{}rcl@{}} {BER}=\frac{{\sum}_{i=1}^{P_{w}} {\sum}_{j=1}^{Q_{w}}[(W[i,j]- W^{\prime}[i,j])^{2}]}{P_{w} \times Q_{w}}. \end{array} $$

The BER value lies between 0 and 1. The watermark extraction is considered perfect if the BER is 0. In such a case, the extracted watermark bits are identical to the embedded/original watermark bits. In contrast, the BER value of 1 indicates a total mismatch between the former and the latter [18, 19]. The symbols in (11) are similar to the ones in (10) i.e. *W* and $W^{\prime }$ stand for the original and extracted watermarks of dimensions *P*_*w*_ and *Q*_*w*_, respectively. Note, the simulation results in Fig. 7 are obtained from the test image: Lena.

### Imperceptibility and capacity analysis

The capacity in Table 2 is measured as the total number of bits embedded within the host image. In the proposed method, it is calculated as a sum of the capacities of the presented signature and fingerprint watermark embedding schemes, respectively. For instance, when the host image of 512 × 512 pixels in size is decomposed to three levels of DWT, the size of the extracted *LH1*, *LH2* and *LH3* subbands is 256 × 256, 128 × 128 and 64 × 64, respectively. Firstly, the LH1 subband is used for the signature watermark embedding via path-1 of the proposed method. Subsequently, the *LH1-DWT* coefficients are divided into 8x8 non-overlapping blocks, producing a total of 1024 blocks. The DCT is then performed on each of these 8x8 blocks and the DCT coefficients are extracted. A pair of DCT coefficients in each of these blocks can be manipulated to embed a signature watermark bit; therefore, a maximum of 1024 watermark bits can be embedded, which is the capacity of the proposed signature watermarking scheme. Secondly, the *LH3* subband, which is 64 × 64, i.e. 4096 coefficients in size, is used within path-2 of the proposed method and is further sub-divided into 2 × 2 blocks, yielding a total of 1024 blocks, which can accommodate no more than 32 × 32 i.e. 1024 watermark bits. Finally, if the watermark size is larger than 32 × 32, the *LH2* subband can then be used for the fingerprint watermark embedding. The *LH2* subband, 128 × 128 in size, can accommodate up to 64 × 64 i.e. 4096 watermark bits. Therefore, the total capacity of the proposed dual watermarking scheme is 5120 bits.

In Table 2, the PSNR value of not available (N/A) is associated with method [15]. The maximum number of grey tones (in the context of multitoning) that method [15] can generate a watermarked image with is 4, whereas the working illustrations of the proposed method are shown by its ability to operate with 4, 8 and 16 grey tones, respectively. The only PSNR value associated with method [15] in Table 2 corresponds to the watermarked image it generated using 4 grey tones. Moreover, the method [15] has not demonstrated its ability to work with 8 and 16 grey tones in its companion paper; therefore, the relevant PSNR values for 8 and 16 grey tones are marked as “N/A” in Table 2. Such analysis not only highlights the contribution of the proposed method but also achieves a fairer comparison with method [15].

**Fig. 8:**
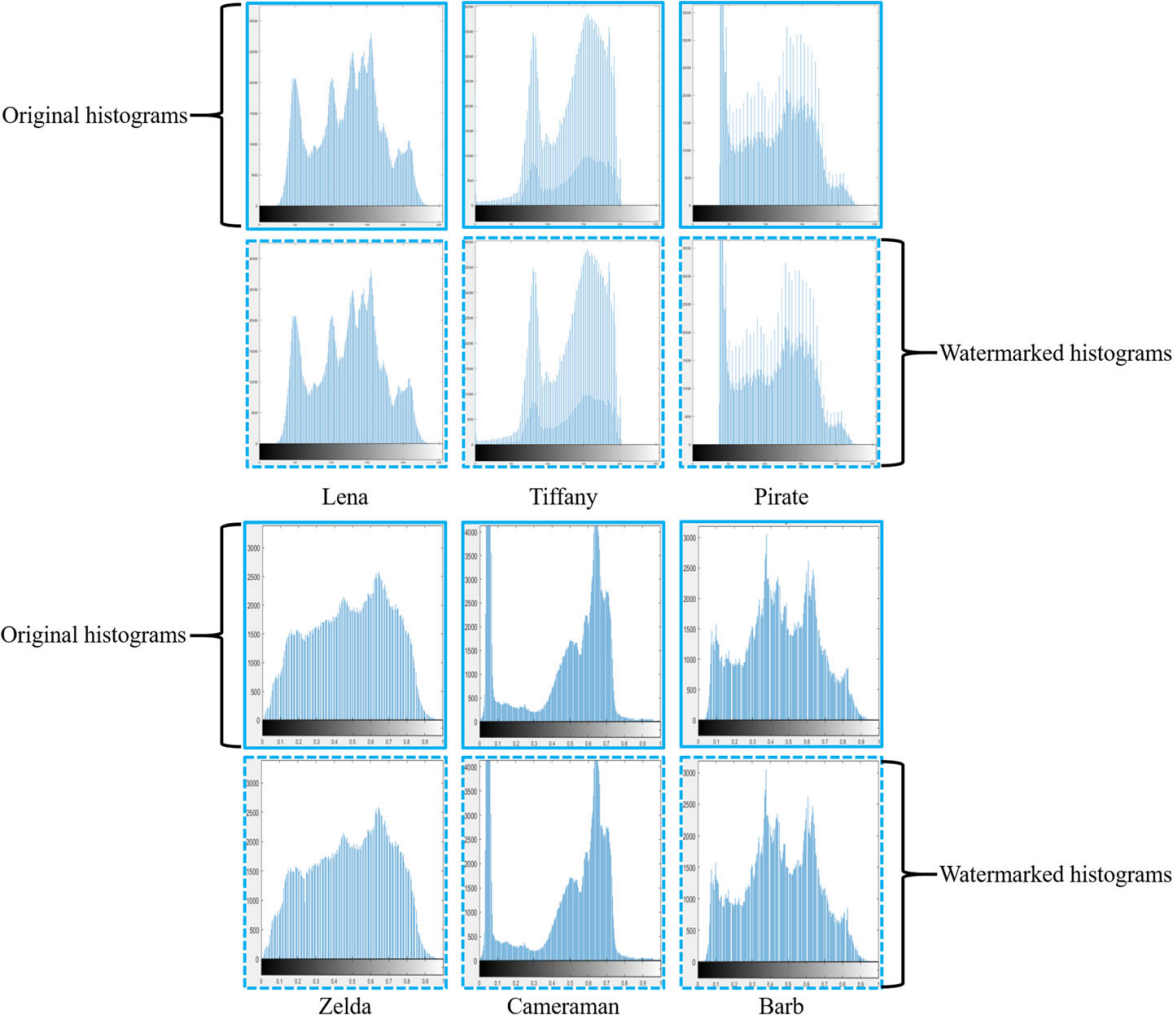
Histogram comparison of the continuous-tone host images (solid boundaries) with the watermarked images (dashed boundaries). Best viewed when zoomed in

**Fig. 9:**
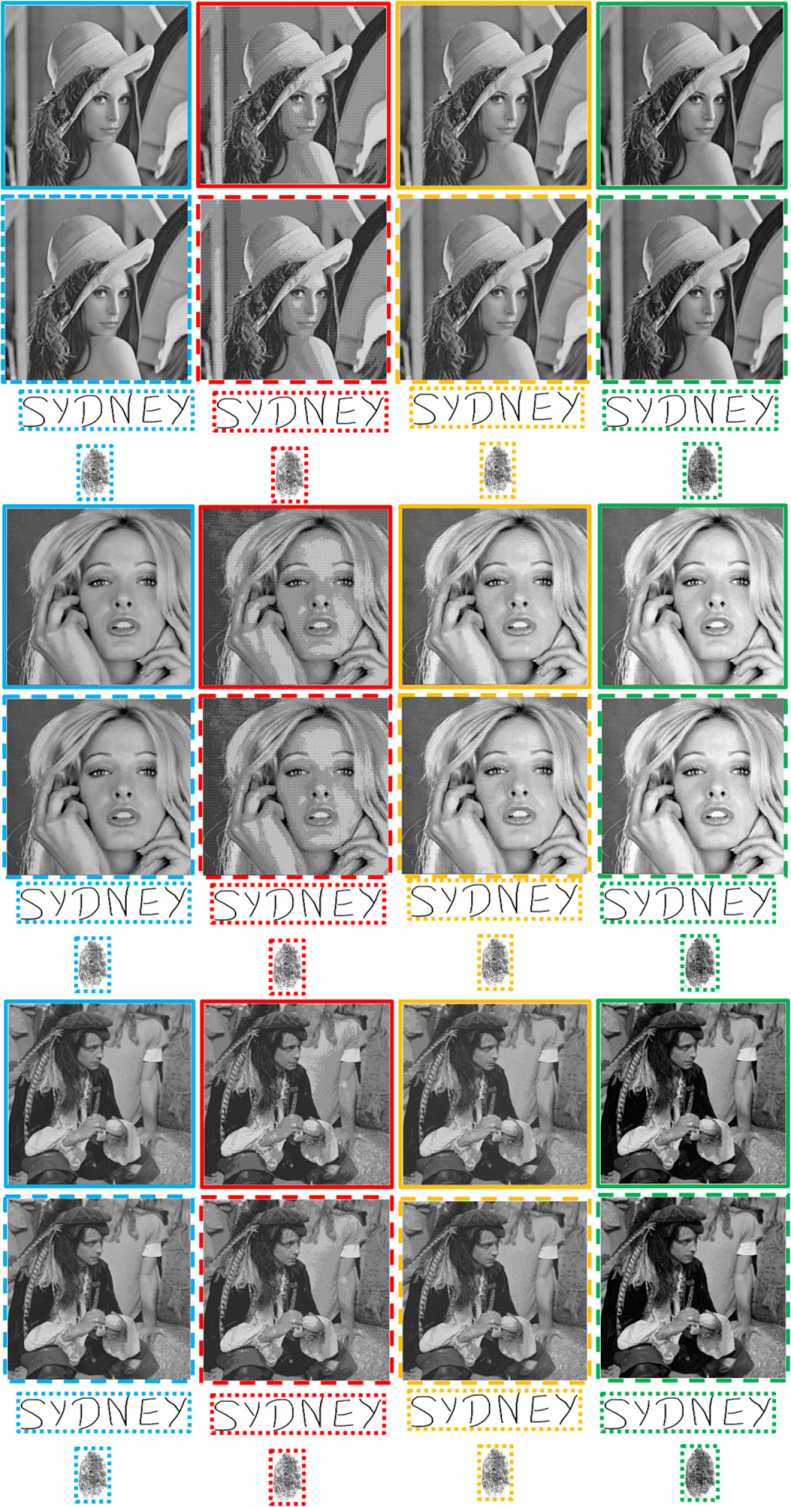
Imperceptibility comparisons of the proposed scheme in the absence of an attack. The un-watermarked (original) continuous-tone images are in the solid sky-blue boundaries. The watermarked continuous-tone images and successively extracted watermarks are in dashed and dotted sky-blue boundaries, respectively. Similarly, the un-watermarked multi-tone images with 4, 8 and 16 grey tones are in the solid red, yellow and green coloured boundaries. Subsequently, the watermarked multi-tone images and successively extracted watermarks are in their corresponding colored (red, yellow and green) dashed and dotted boundaries, respectively. Best viewed when zoomed in

**Fig. 10:**
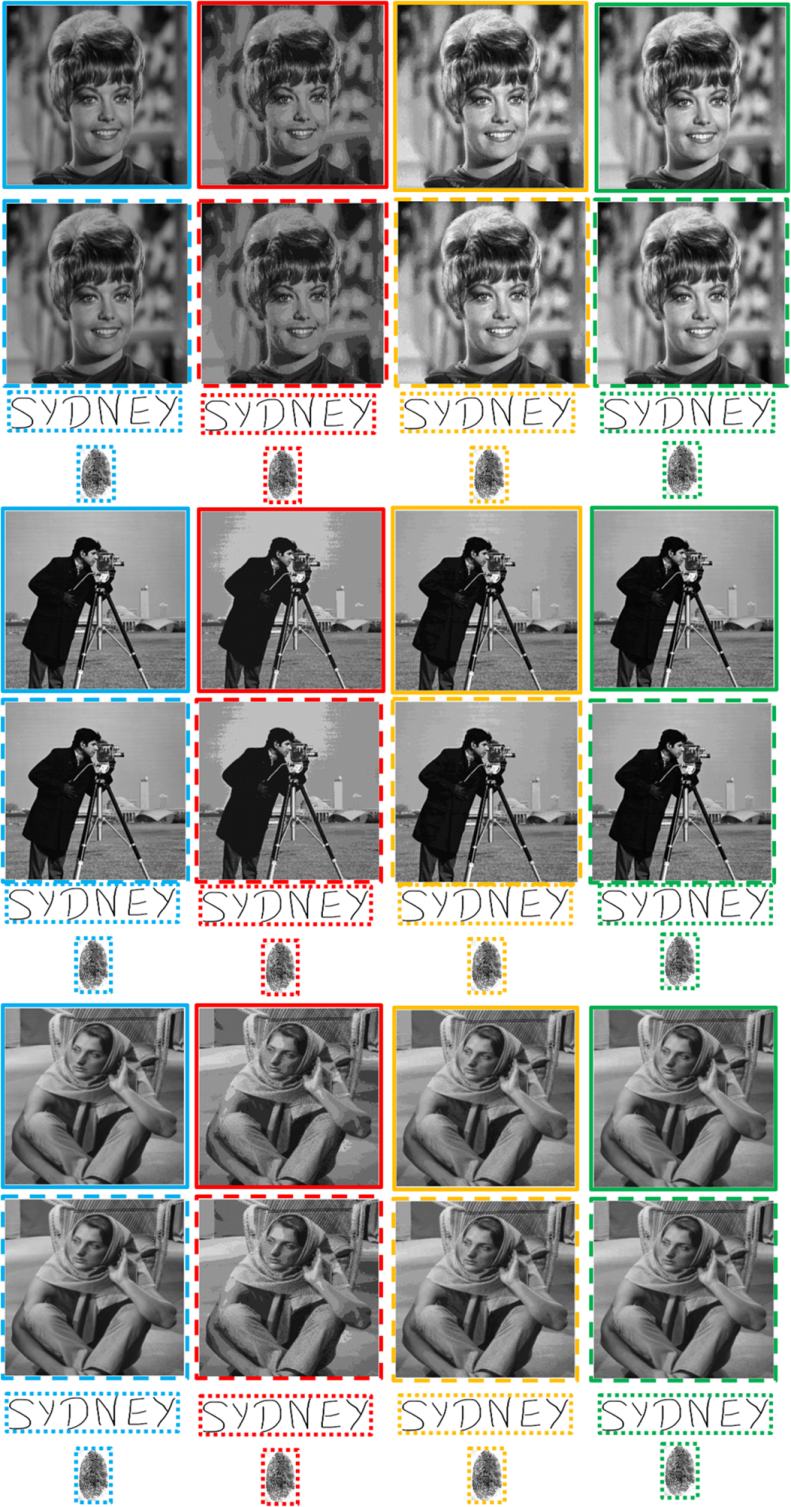
More imperceptibility comparisons of the proposed scheme in the absence of an attack. The un-watermarked (original) continuous-tone images are in the solid sky-blue boundaries. The watermarked continuous-tone images and successively extracted watermarks are in dashed and dotted sky-blue boundaries, respectively. Similarly, the un-watermarked multi-tone images with 4, 8 and 16 grey tones are in the solid red, yellow and green coloured boundaries. Subsequently, the watermarked multi-tone images and successively extracted watermarks are in their corresponding colored (red, yellow and green) dashed and dotted boundaries, respectively. Best viewed when zoomed in

Firstly, in terms of the continuous-tone images, Table 2 shows that the method in [4] is surpassed by every other method in terms of both imperceptibility and capacity. Secondly, although the method in [53] is outperformed by the methods in [20] and [35] in imperceptibility, its capacity attribute is superior to its counterparts and is on par with the proposed method. Third, the method in [20] is superior in imperceptibility when compared with the method in [35], however, the latter has better capacity because it is capable of embedding four watermarking bits in an 8 × 8 block, whereas the former embeds only two. In the same table, it can also be observed that the method in [24] has the highest capacity and is capable of achieving the third-highest PSNR value. The proposed watermarking scheme outperforms all of these methods in terms of imperceptibility. Furthermore, it also higher in capacity than [53], thus, surpassing methods in [4, 20] and [35] in this context as well. Numerically, the proposed method is superior to the other methods shown in [4, 20, 24, 35, 53] from the imperceptibility viewpoint by 9.29%, 6.8%, 5%, 8.69%, 5.53%, respectively. Finally, the authors in [21, 22] have used histograms to prove the effectiveness of their embedding strategies. Similarly, the histogram comparisons in Fig. 8 suggest a cumulative resemblance of 98.4 % between image pairs. Subsequently, the histogram pair of the test image Tiffany has the least histogram similarity i.e. 97.9 %, whereas, the highest similarity is attained by Lena with 98.6%. This is an indication of a successful embedding strategy as the processed images are indistinguishable from the host images to the HVS. A comparison between the original/host images and the watermarked images without an attack is illustrated via Figs. 9 and 10. Subjectively, it can be noticed that watermarked images (both continuous-tone and multi-tone) contained within the dashed boundaries appear to be serene and homogeneous in tone. Consequently, the watermarked image displays a smooth transition between grey levels and as a result is imperceptible to the HVS.

**Fig. 11:**
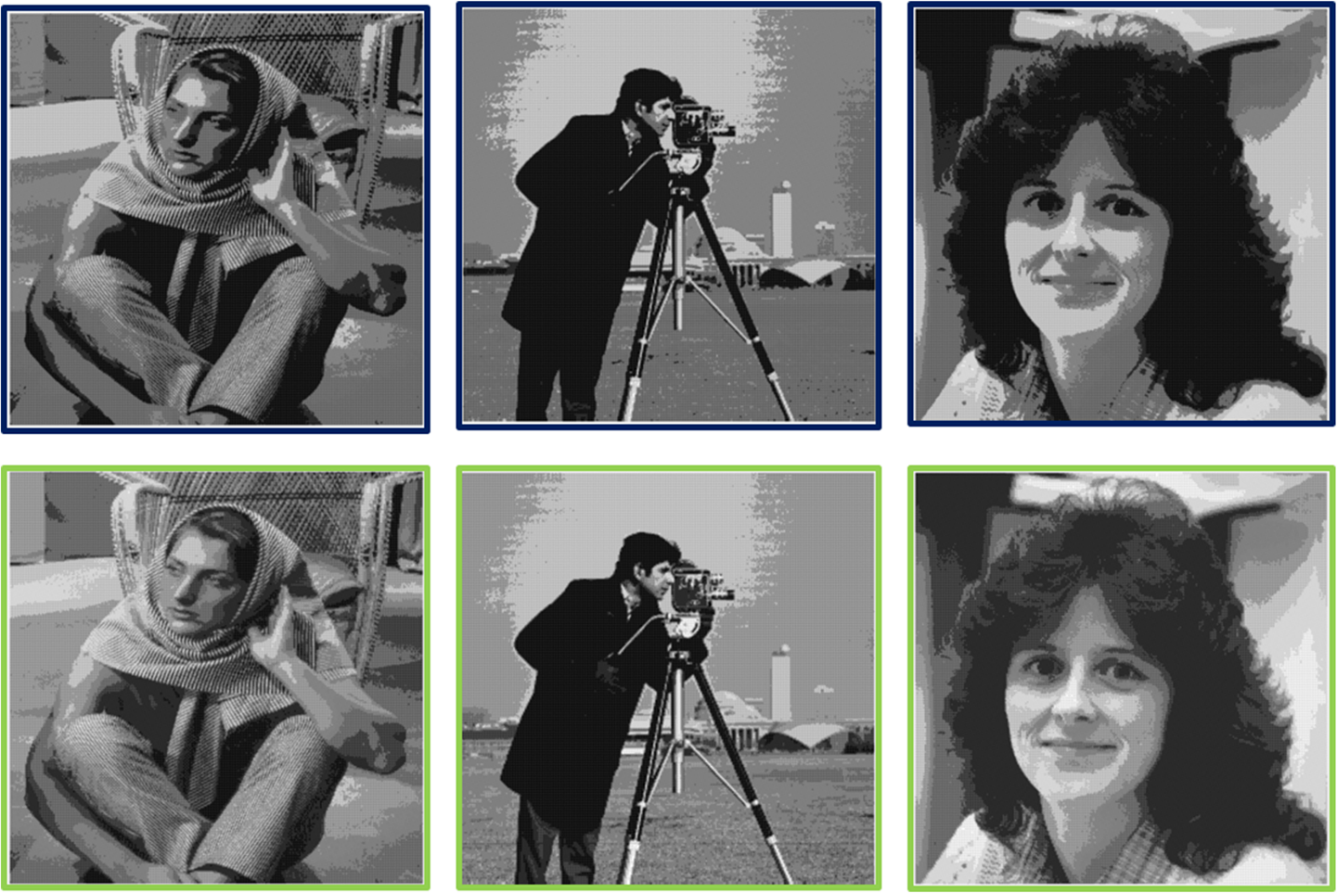
Imperceptibility comparisons of the watermarked multi-tone image produced using the ED multitoning method in [43] with the proposed multi-tone scheme (bottom row). Illustrated images are comprised of 4 grey levels. Best viewed when zoomed in

Similarly, in terms of the multi-tone image analysis, Table 2 shows that the OD-based methods in [15] and [46] have the same capacity, but the latter performs better with respect to imperceptibility. The same table also illustrates that the former is able to achieve only 4 tones of grey, whereas the latter method, like the proposed method, is successfully able to handle more tones of grey. Table 2 illustrates that the proposed method outperforms its counterparts with regard to imperceptibility. The improvement in PSNR values of the proposed method against the methods shown in [15] and [46], while operating at 4 grey tones, is 2.1% and 1%, respectively. Likewise, the PSNR improvement over [46] at 8 and 16 grey levels is 1.77% and 1.63%, respectively. Table 2 also compares the proposed method with other state-of-the-art ED-based methods [9, 63]. At 4 grey tones, ED-based methods outperform the method in [15] with respect to both capacity and imperceptibility. However, as quantization levels increase, ED-based methods can produce severe artefacts, degrading the imperceptibility. Readers are referred to [48] as it covers both subjective and objective assessments of multitoning using ED and OD. Finally, the simulation results of the images produced using the ED-based multitoning method by Sarailidis et al. [43] are compared with the images achieved by the proposed multitoning strategy in Fig. 11. The top row of Fig. 11, shows that the imperceptibility of the ED-based images is compromised, and artefacts are prominent to the observer.

### Robustness/Security analysis

**Fig. 12:**
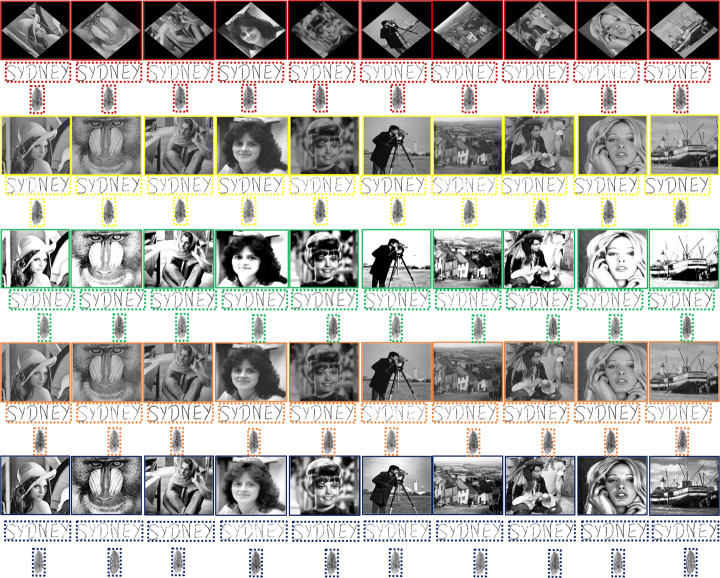
Robustness/Security comparisons of the proposed scheme on continuous-tone images under various attacks. The solid red, yellow, green, orange and blue boundaries contain the watermarked images under the rotation attack at 45, Gaussian Noise at 0.001 and gamma correction at 0.75, Salt & Pepper noise at 0.02 and histogram equalization, respectively. All dashed boundaries represent the extracted watermarks from the attacked watermarked images. Best viewed when zoomed in

The continuous-tone watermarked images under various StirMark attacks (available at [42]) and the extracted watermarks are shown in Fig. 12. Table 3 compares the proposed method with the state-of-the-art blind and non-blind methods. The NCC values in Tables 3 and 4 demonstrate the similarity between the embedded and extracted watermarks, respectively. Note, in Tables 3 and 4, whenever a state-of-the-art method has not covered a specific attack that’s been tested by the proposed method, the former is given with the NCC value of “N/A”. It signifies that the state-of-the-art method has not analysed the security/robustness attribute under a particular attack. Such analysis at first highlights any shortfalls within a state-of-the-art method and then justifies the proposed method’s contribution in bridging those shortfalls.

**Fig. 13:**
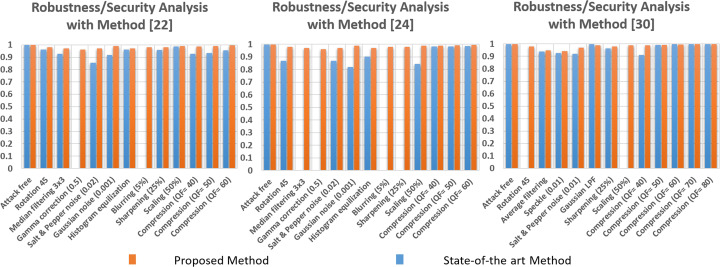
Robustness/Security analysis of the proposed method and its comparison with other state-of-the-art methods in [22, 24, 30]. Watermarking attacks are denoted by the *x-axis* and the NCC values are represented by the *y-axis*. Best viewed when zoomed in

Table 3 illustrates that the method in [4] lacks in terms of the robustness/security evaluations. The method in [4] is blind and targets continuous-tone images. It is tested for its resistance against the rotation attack, in which it outperforms its peers in Table 3 and is on par with the proposed method. In contrast, its performance under scaling attack is surpassed by every other method in the same table. The method in [53] is outperformed by its counterpart methods [20, 35] with respect to overall/average NCC value. However, apart from the proposed method, it outperforms every other method under the compression attack when performed at different QF values. Similarly, the method in [35] is better in the average NCC value than methods in [4, 53]. It is also more resilient to the rotation attack when compared to the method in [20], although the latter is better in overall performance by 2.3%. It is to be noticed that the non-blind methods [1, 3, 33, 47] in Table 3 attain higher average NCC values in comparison to the blind methods. This signifies that the watermark reconstruction rate is higher in the non-blind methods, thus, making them more resilient to watermarking attacks than their blind counterparts. Lastly, the proposed method is as skillful as the method in [20] under the Gaussian noise and scaling attacks. Moreover, it outperforms the latter and other methods in Table 3 with respect to the average NCC value, thus, making it superior in overall robustness. The proposed method is 10.3%, 1.74%, 3.07%, 6.77% better than the methods in [4, 20, 35, 53], respectively. Finally, due to space constraints, Table 3 is incapable of comparing the proposed method with methods in [22, 24, 30]. These methods are state-of-the-art as well as the extensions of the methods in [20, 35, 53], respectively. To this end, Fig. 13 is employed to provide more perceptual analysis and graphically compare the proposed method with methods in [22, 24, 30] regarding the NCC values.

The proposed watermark embedding and extraction/detection are performed in the transform domain and the geometrical attacks are performed in the spatial domain. To this end, it’s highly unlikely for the geometrical attacks to eliminate all of the frequency components representing the watermark information. Hence, in the literature, this is one of the main reasons the transform domain-based methods are preferred for robust watermarking over the pixel/spatial domain-based methods [22, 24, 25, 41].

**Fig. 14:**
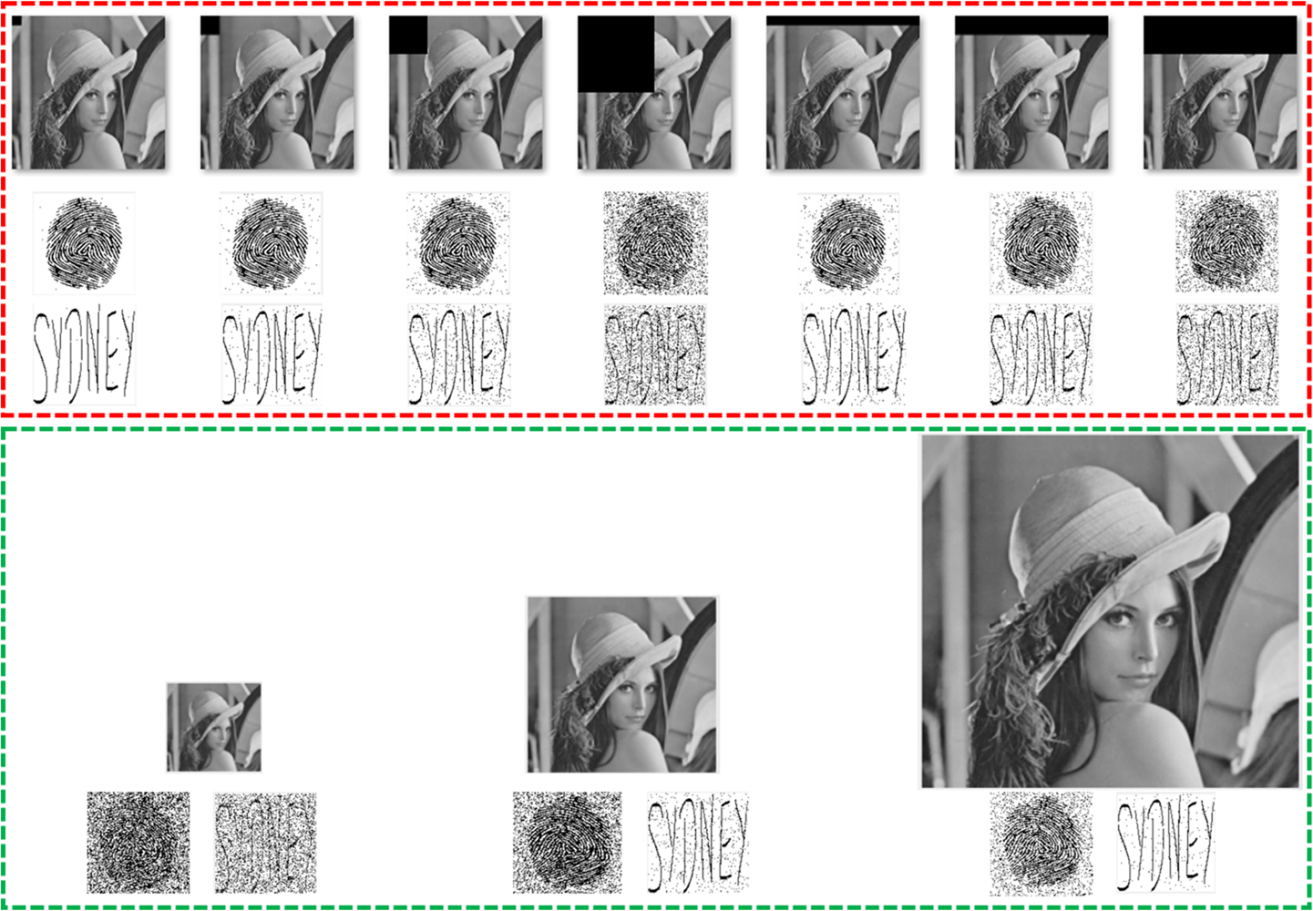
The red boundaries cointain the cropped images as well as the extracted watermarks. The green boundaries contain the resized images and the extracted watermarks. Best viewed when zoomed in

Concerning the geometrical attacks, the watermark detection/extraction in the proposed method is achievable after the cropping and the resizing attacks. The working illustrations of the proposed method for these attacks are given below in Fig. 14.

**Fig. 15:**
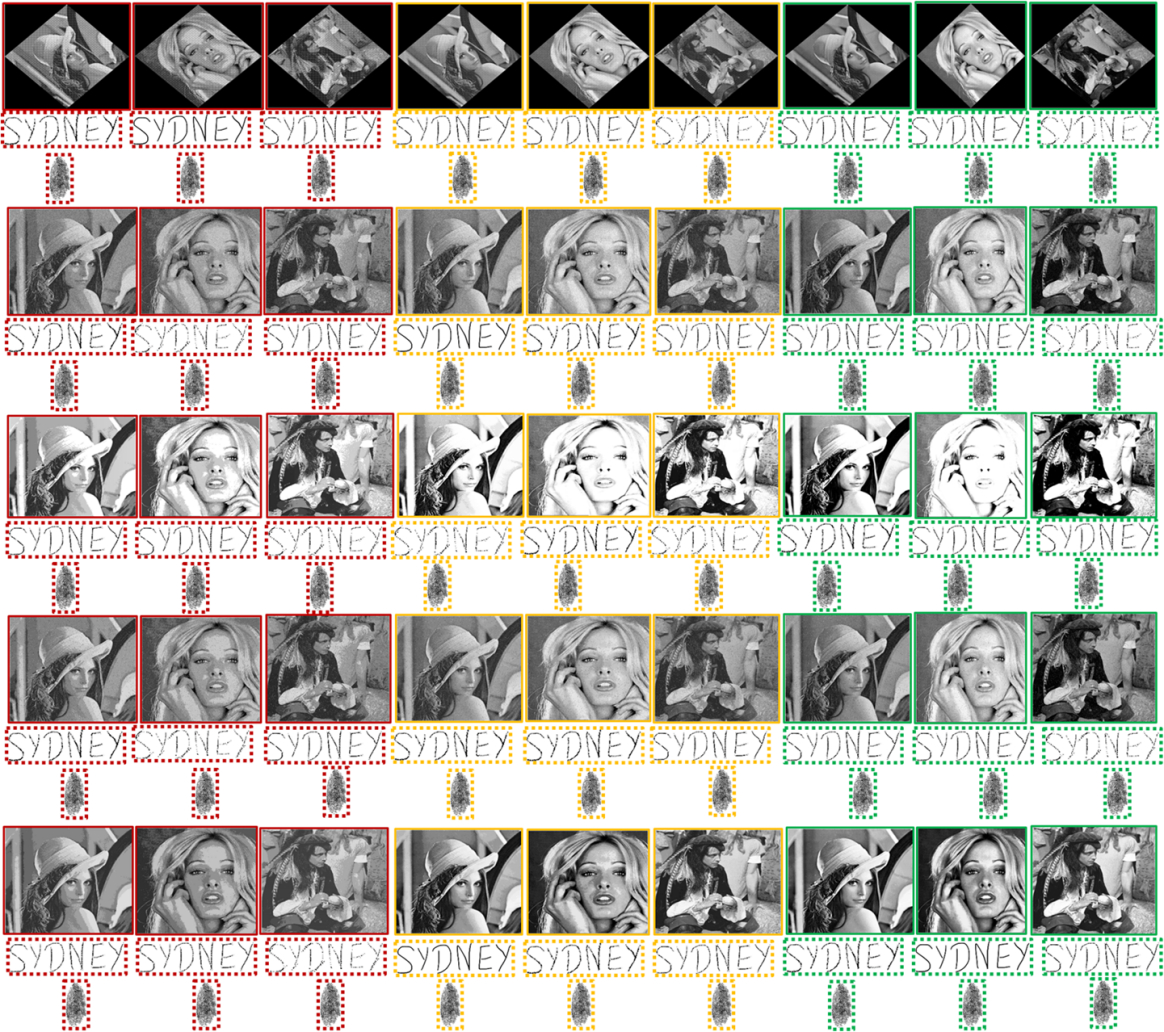
Robustness/Security comparisons of the proposed scheme on the multi-tone images under various attacks. Solid red, yellow and green boundaries contain the watermarked images composed of 4, 8 and 16 grey tones, respectively. Successively, the extracted watermarks from these images are contained within their corresponding coloured dotted boundaries. Best viewed when zoomed in

**Fig. 16:**
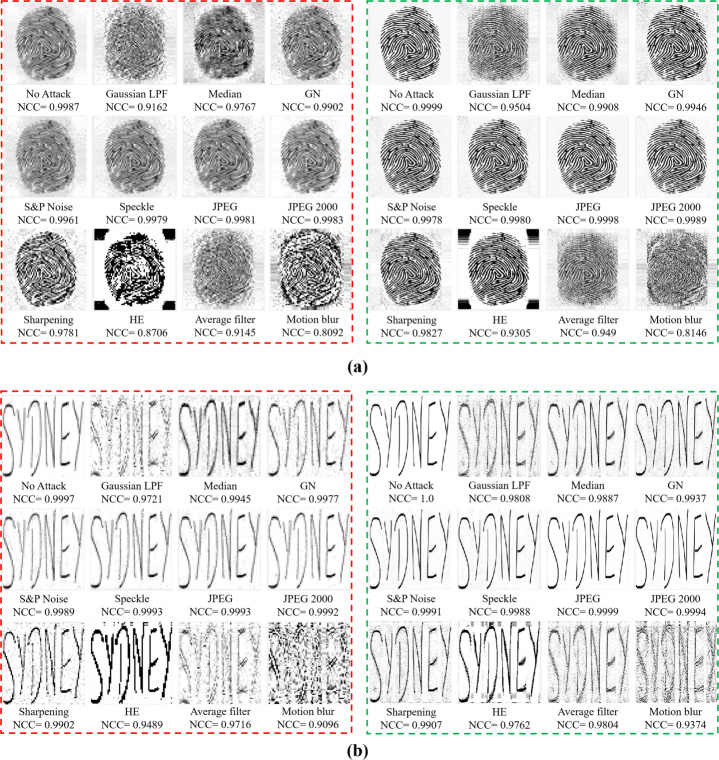
Robustness/Security comparisons of the extracted watermarks under different attacks. (a) The extracted fingerprint watermarks after being processed by the ED-based multitoning method [43] are within the dashed red boundary. In contrast, the ones processed by the proposed method are in the dashed green border. (b) The extracted signature watermarks after being processed by the ED-based multitoning method [43] are within the dashed red boundary. In contrast, the ones processed by the proposed method are in the dashed green boundary. Best viewed when zoomed in

Likewise, the proposed method’s robustness performance on multi-tone images is illustrated via Table 4 and Fig. 15. Firstly, Table 4 shows that the method in [15] is outperformed by the method in [46] with respect to the average NCC value while operating at 4 grey tones. In the same context, the method [46] is surpassed by the proposed method operating at 4, 8 and 16 grey tones, respectively. Secondly, the proposed method has also shown an improvement with respect to the average NCC value when compared with the method in [15] whilst operating at 4 grey tones. Finally, the robustness/security of the watermarks extracted from the multitoned watermarked images achieved via ED-based method [43] and the proposed method are compared in Fig. 16. In Fig. 16a, the extracted fingerprint watermarks after being processed by the ED-based multitoning method [43] are within the dashed red boundary, whereas the ones processed by the proposed method are in the dashed green boundary. Similarly, Fig. 16b compares the signature watermarks after being processed by the ED-based multitoning method [43] are within the dashed red boundary. In contrast, the ones processed by the proposed method are in the dashed green boundary. Figure 16 illustrates the superiority of the proposed method over the method [43] from both objective and subjective viewpoints.

### Processing time analysis

The processing time (PT) of the proposed scheme is dependent on the size of the host image and that of the watermark itself. The larger are these sizes the longer is the processing time. Furthermore, in a watermarking process the total processing time, is measured as a sum of the individual times taken by the embedding (*Time*_*Embedding*_) and the extraction (*Time*_*Extraction*_) processes, respectively. The time consumed by the proposed watermarking process in case of continuous-tone image (*PT*_*Continuous*_) is calculated via (12). Successively, in case of a multitoned image, the processing time (*PT*_*Multitone*_) is calculated as per (13), where *Time*_*Multitoning*_ is the time taken in generating a multi-tone image.

12 $$ \begin{array}{@{}rcl@{}} PT_{Continuous}=Time_{Embedding}+Time_{Extraction} \end{array} $$

13 $$ \begin{array}{@{}rcl@{}} PT_{Multitone}=PT_{Continuous}+Time_{Multitoning} \end{array} $$

The objective evaluation of the proposed method’s PT and its comparison with other methods is presented within Table 5. As per the above discussion, the total processing time in Table 5 is defined as a sum of the embedding time and the extraction time. To this end, whenever a state-of-the-art method has not covered the timings of either of these components, it is assigned with a value of “N/A”. The given table shows that in continuous-tone images, the method in [4] has the highest PT, whereas, the method [24] has the lowest. The proposed method has outperformed every other method in Table 5, apart from the method in [24]. Moreover, in multi-tone images, the proposed method and every other is outperformed by the method in [15]. However, it is unable to operate beyond 4 grey levels. Unlike its counterpart ED-based methods [9, 63], the proposed method and others [15, 46] in Table 5 use OD to achieve multitoning. Consequently, the latter methods are faster as ED employs a feedback loop for quantization error calculations. The calculated error is successively pushed forward to four neighbouring pixels and this distribution continuous until the culmination of the multi-tone image. This imposes an overhead of additional multiplications and additions/subtractions, making ED-based methods expensive in PT.

## Conclusion

This paper presents a novel dual watermarking approach for identity protection. Firstly, the proposed watermarking scheme uses the owner’s signature and fingerprint as watermarks through which the ownership and validity of the media can be proven and kept intact. Consequently, in terms of capacity, the proposed scheme is on par with the existing state-of-the-art methods. Secondly, the proposed embedding approach uses DWT, DCT and a novel median-based embedding block selection procedure. Working in conjunction, these techniques enable the proposed watermarking scheme to outperform existing methods in terms of imperceptibility and robustness/security. Thirdly, the application versatility of the proposed scheme is demonstrated by its ability to operate on both the continuous tone/greyscale images and the multitoned images. The former are intensively used in the electronic media, whereas the latter is a widely adopted strategy in modern printing. Finally, the proposed dual watermark scheme objectively outperforms the existing state-of-the-art methods in terms of imperceptibility and robustness/security attributes, measured by PSNR and NCC values, respectively.

The proposed image watermarking strategy employs a pair of robust watermarks, which provides excellent copyright protection. In the future, the proposed scheme could be improvised to achieve multiple goals of copyright protection and authentication. Such a scheme will be embedded with two watermarks; one will be robust to provide copyright protection, and the other will be fragile to facilitate the authentication capability. Moreover, in the case of tampering, the fragile watermark will also exhibit tamper detection and tamper localisation abilities. To this end, the experimental analysis will also be extended to include parameters, such as the false positive rate (FPR) and the false-negative rate (FNR), used for measuring the accuracy of the tamper detection and the tamper localisation attributes, respectively.
